# Supplementary material for: Silver Carp (Hypophthalmichthys molitrix) (Asian Silver Carp) Presence in Danube Delta and Romania—A Review with Data on Natural Reproduction
Source: Life (Basel). 2022 Oct 12;12(10):1582. doi: 10.3390/life12101582 (PMC9605455; doi:10.3390/life12101582)
Supplement: Supplementary file 1 [file life-12-01582-s001.zip › life-1926587-supplementary.pdf]

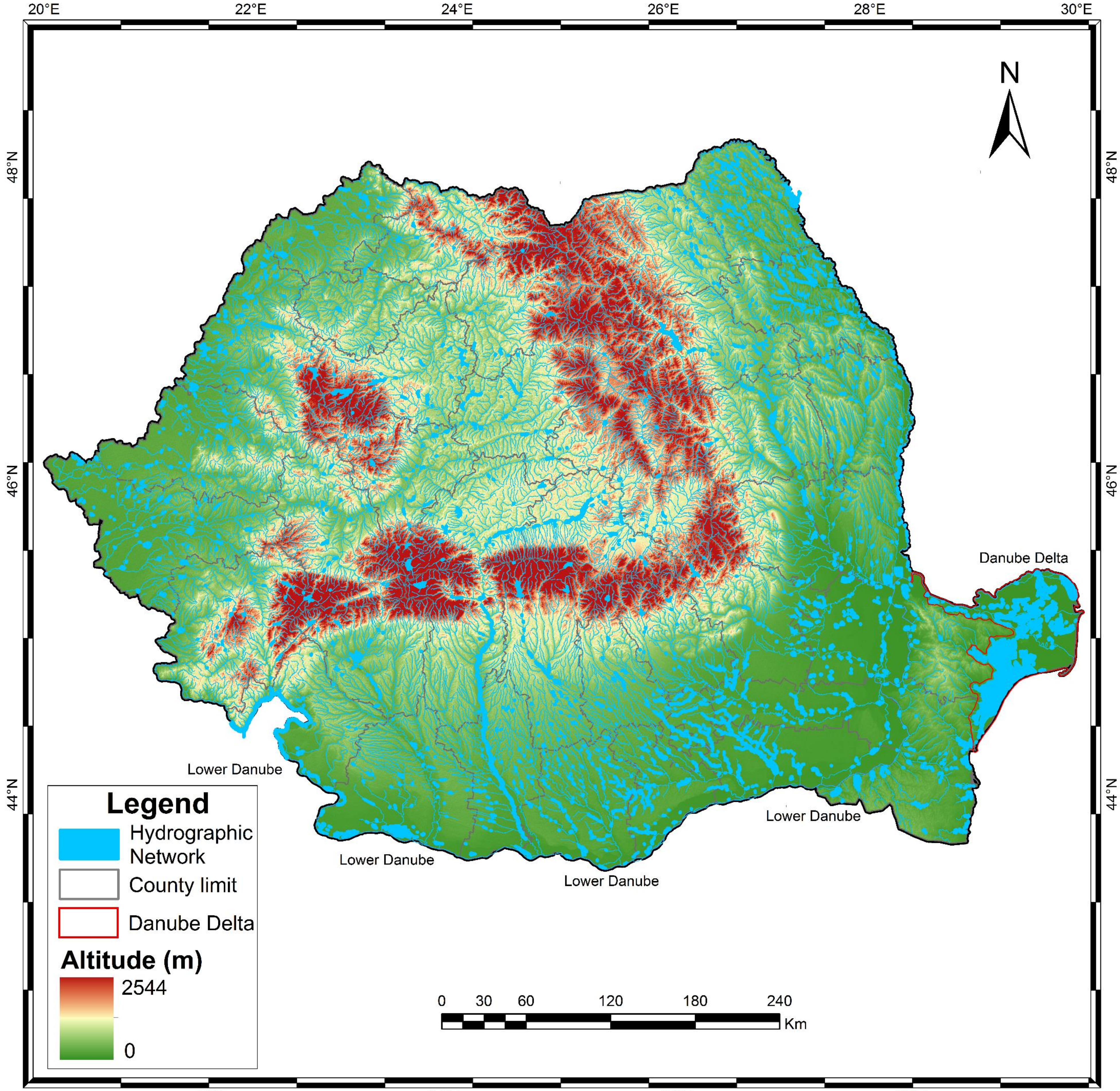

Supplementary Figure S1. Map of the hydrographic network of Romania with emphasis on the lower course of the Danube and the Danube Delta
